# Efficient organogenesis and taxifolin production system from mature zygotic embryos and needles in larch

**DOI:** 10.48130/FR-2023-0004

**Published:** 2023-02-23

**Authors:** Xuetong Yan, Kejia Wang, Keyuan Zheng, Lifeng Zhang, Yang Ye, Liwang Qi, Mulan Zhu

**Affiliations:** 1 National Key Laboratory of Plant Molecular Genetics (NKLPMG), CAS Center for Excellence in Molecular Plant Sciences, Shanghai 200032, China; 2 Shanghai Key Laboratory of Plant Functional Genomics and Resources, Shanghai Chenshan Botanical Garden, Shanghai 201602, China; 3 State Key Laboratory of Tree Genetics and Breeding, Research Institute of Forestry, Chinese Academy of Forestry, Beijing 100091, China; 4 State Key Laboratory of Drug Research and Department of Natural Products Chemistry, Shanghai Institute of Materia Medica, Chinese Academy of Sciences, Shanghai 201203, China

**Keywords:** *Larix olgensis*, Mature zygotic embryo, Needle, Organogenesis, Taxifolin production

## Abstract

The deciduous conifer larch has been widely distributed around the world, is a high-quality wood species and is also used to extract industrial raw materials and medicines. In this study, we developed an organogenesis protocol for *Larix olgensis* from both mature zygotic embryos and needles, and analyzed the content of taxifolin in different tissues. The highest callus induction (96.8%) from mature zygotic embryo was found in the Douglas-fir Cotyledon Revised (DCR) medium augmented with 2.0 mg·L ^−1^ 6-Benzylaminopurine (6-BA) and 0.2 mg·L ^−1^ α-Naphthaleneacetic acid (NAA), while from needles the highest callus induction (92.03%) was found in the Murashige and Skoog (MS) medium augmented with 3 mg·L ^−1^ 6-BA and 0.3 mg·L ^−1^ NAA. The best shoot regeneration capacity from zygotic embryo-derived calli (83.3%) was obtained in DCR medium augmented with 1.0 mg·L ^−1^ 6-BA and 0.01 mg·L ^−1^ NAA, and needle-derived calli were 77.3%. The shoots achieved the highest elongation (75.6%) in the DCR medium supplemented with 0.5 mg·L ^−1^ 6-BA, 0.05 mg·L ^−1^ NAA and 2 g·L ^−1^ activated charcoal (AC). The rooting rate was 62.8% in DCR medium augmented with 3 mg·L ^−1^ Indole-3-butyric acid (IBA) and 100 mg·L ^−1^ phloroglucinol (PG). The accumulation of the taxifolin in elongation shoots and lignified elongation shoots have greatly improved along with the development process, were 28.6 µg·g ^−1^, and 53 µg·g ^−1^ respectively. The content of the taxifolin in callus was 1.99−5.26 µg·g ^−1^, adventitious shoots were 4.8 µg·g ^−1^, and adventitious roots were 2.86 µg·g ^−1^. We report an efficient organogenesis and taxifolin production protocol in larch for the first time.

## INTRODUCTION

The deciduous conifer *Larix olgensis* (Henry), also known as Changbai larch, is mainly distributed in temperate mountainous areas of the northern hemisphere. This species is highly valued in forestry production due to its good adaptability to the environment and short rotation periods in plantations, which also plays an important role in the maintenance of the mountain environment and the construction of the mountain landscape ^[ 1] ^. The monomolecular fibers of larch wood are long and have the advantage of corrosion resistance and pressure resistance, making it a high-quality building material ^[ 2] ^, and the basic raw material for high-grade printing paper. Taxifolin and arabinogalactan are two important metabolites that exist in the xylem of larch, which have a wide range of applications in medicine, food, health care products, and other industries. Recently, these two ingredients have been permitted to be used as food additives.

With the increasing shortage of forest resources in the world, there is a large market for high-quality larch breeding. The genetic improvement of larch has received much attention. However, larch species have high heterozygosity, large progeny variability, long breeding cycle, and slow effect of trait improvement. Traditional breeding methods are difficult to achieve directional trait improvement. At present, the main propagation method for larch plantation is seedling cuttings. Therefore, it is very urgent to establish an efficient *in vitro* regeneration system in larch. The *in vitro* regeneration technology has many excellent characteristics, such as production efficiency (a large number of seedlings obtained in a short time) and drastically shortened breeding time. The whole process is stable and reliable with strong controllability ^[ 3] ^, since it is not affected by external conditions. Therefore, the *in vitro* regeneration technology has been widely used in large-scale breeding of high-quality seedlings, genetic transformation, and gene editing.

There have been some reports on the *in vitro* regeneration of larch, such as *Larix sibirica* ^[ 4] ^, *Larix gmelinii* ^[ 5] ^, *Larix gmelinii* var *. principis-rupprechtii* ^[ 6] ^, *Larix kaempferi* ^[ 7] ^, *Larix olgensis* ^[ 8]^ and hybrid larch ( *Larix kaempferi* × *Larix gmelinii *) ^[ 9]^ etc. Most of them focus on somatic embryogenesis studies, but it is usually along with deformed embryos and seedling problems, which is far from the 80%−85% germination rate of somatic embryos in commercial application requirements ^[ 10] ^. Besides, there are fewer studies on the *in vitro* regeneration system of larch organogenesis from *Larch gmelinii* mature embryos, old tree shoots ^[ 11, 12] ^, western American larch mature embryos ^[ 13] ^, hybrid larch ( *Larix* × *Eurolepis* Henry) shoots ^[ 14] ^, European larch shoots ^[ 15] ^. In these mentioned reports, a certain number of adventitious buds and a small amount of intact regenerated plants were obtained *via* the callus route, but problems such as low callus differentiation efficiency, slow elongation, difficult rooting, and low proliferation efficiency are still concomitant, which inhibited the subsequently commercial application. Although *in vitro* regeneration with leaf explants have been reported in other woody species, such as *Artemisia annua* ^[ 16] ^, *Robinia pseudoacacia* ^[ 17]^ etc, organogenesis *via* callus from needles is rarely reported in conifers. Obviously, a complete and efficient regeneration system for larch is lacking. In addition to the rapid propagation of plants based on the established organogenesis system, the plant tissues have artificially regulated potential in biological efficacy. In recent years, the advantages of tissue culture in the production of pharmaceutical ingredients are gradually realized ^[ 18, 19] ^. For example, the content of secondary metabolites could be induced to a higher level through tissue culture ^[ 20] ^, thereby promoting its large-scale medicinal use and maximizing the medicinal value of the plant ^[ 21] ^. Taxifolin is believed to exist in the xylem of the rhizomes of larch. Some researchers established a callus induction system for taxifolin extraction by using the branches of larch as explants ^[ 22] ^, but the experiment stopped in the callus induction without subsequent differentiation process. Thus, the establishment of the production of the active ingredients by *in vitro* regeneration system is very challenging.

In this study, we developed a protocol for the organogenesis of larch using mature zygotic embryos and needles (of *in vitro* regenerated plantlets). The establishment of this efficient *in vitro* regeneration system can not only be used for the supply of plantation seedlings but also provide sustainable alternative medical raw materials without exploiting natural plants, which is of great significance to promoting the social economy and maintaining the ecological environment. To our knowledge, this is the first study of the complete and efficient regeneration system in larch. Moreover, we first report indirect organogenesis using needles as explants in conifer.

## MATERIALS AND METHODS

### Explant material preparation

Seeds of Larix olgensis ( *L. olgensis*) that had been randomly collected from mature and healthy plants from county Shalan, Ning’an (E 128º27' − 128º55' and N 44º02' − 44º20') of Heilongjiang province, China, were provided by the Xiaobeihu Mushulin Forest Farm, China.

After de-husking, the healthy-looking seeds were washed thoroughly with flowing tap water and distilled water and then surface-sterilized in potassium permanganate (1‰ [v/v] for 3 min) ethanol (70% [v/v] for 1 min) and sodium hypochlorite (20% [v/v] for 12 min), followed by five rinses with double-distilled autoclaved water. The endosperm should be removed before embryo explant inoculation.

For needle explants, the seeds were first planted in plastic pots (22 cm in diameter), containing a mixture of autoclaved horticulture soil and perlite in a 2:1 ratio, and maintained within growth chambers (Shanghai Chenshan Botanical Garden, Songjiang district, Shanghai, China) under a 16-h photoperiod (33.73 µmol∙m ^−2^∙s ^−1^ light intensity provided by cool white fluorescent tubes) at a temperature of approximately 25 °C and relative humidity of 80%.

**Figure 1:**
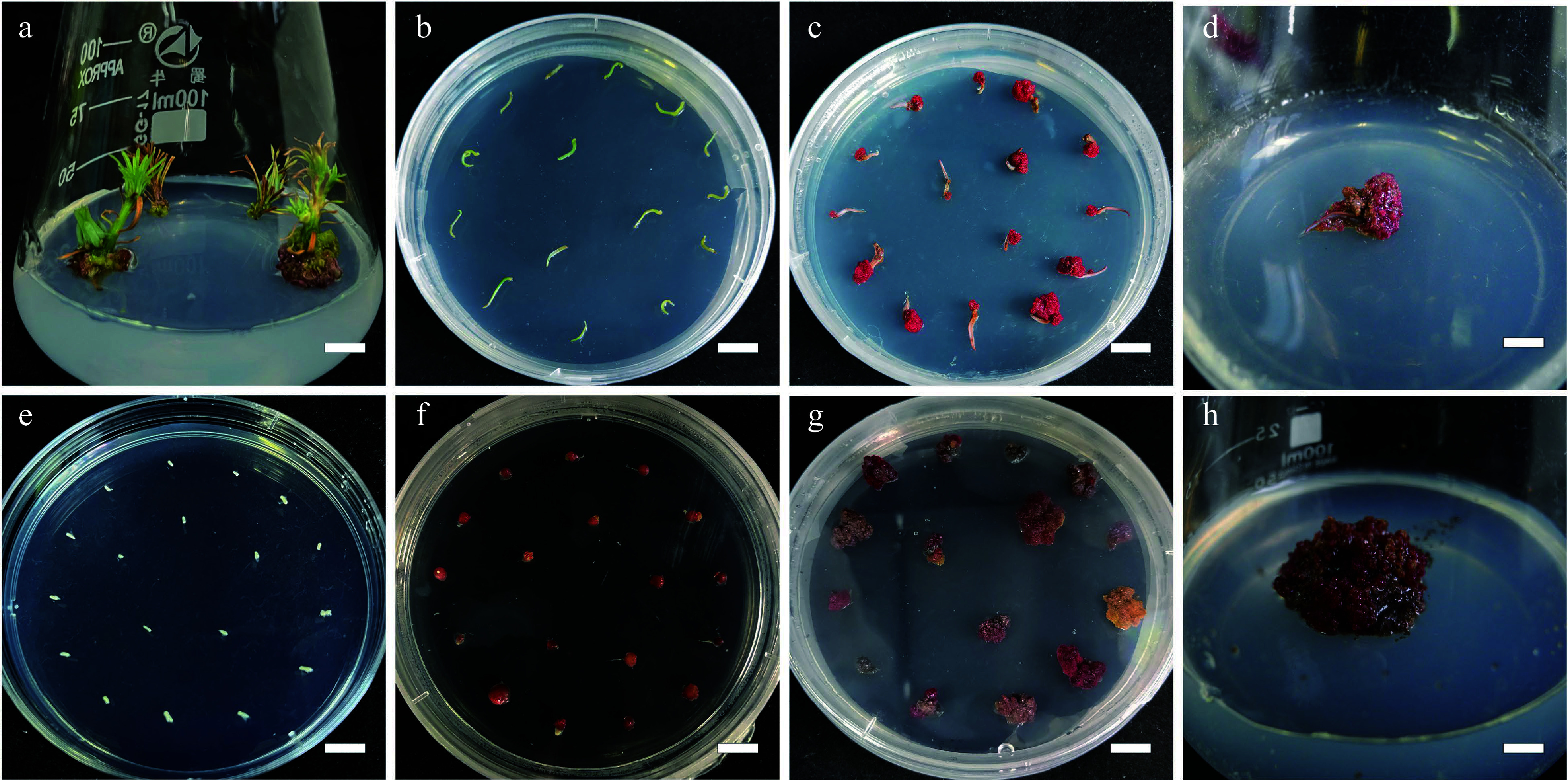
Callus induction from needle explants and mature zygotic embyro explants in *L. olgensis.* (a) Sterilized stem sections for obtaining needle explants, bar = 0.7 cm. (b) Needle explants operated in DCR medium augmented with 3 mg·L ^−1^ 6-BA and 0.3 mg·L ^−1^ NAA, bar = 1 cm. (c) Calli from needle explants generated in 4 w, bar = 1 cm. (d) Calli from needle explants generated in 8 w, bar = 0.6 cm. (e) Sterilized mature zygotic embryo as explants, bar = 1 cm. (f) Calli from mature zygotic embryo explants generated in 3.0 mg·L ^−1^ 6-BA combined with 0.3 mg·L ^−1^ NAA, bar = 1 cm. (g) Calli from mature zygotic embryo explants generated in 4 w, bar = 1 cm. (h) Calli from mature zygotic embryo explants generated in 8 w, bar = 0.6 cm.

After a 2-week growth period of the *L. olgensis* plants ( Fig. 1a, b), shoot tips (10 – 25 mm) were collected and thoroughly washed under running tap water with cleanser essence for approximately 10 min and then transferred to a laminar flow clean bench. The shoot tips of *L. olgensis* were washed again with double-distilled autoclaved water and then surface-sterilized in 70% (v/v) ethanol for 30 s twice, followed by 1% (v/v) benzalkonium bromide for 6 min, and rinsed three times with sterile water. The sterilized *L. olgensis* shoot apices were further cut into smaller pieces (7–15 mm) with sterile scalpels to remove cut end surfaces that were in direct contact with the sterilizing agents. These shoot tips were inoculated in Murashige & Skoog (MS) Medium with B5 vitamins supplemented with 0.1% (v/v) Plant Preservative Mixture (PPM) for pre-culture. After an additional 6 weeks, *L. olgensis* needles were collected from the pre-culture shoot tips, pre-culture needles were used for the *in vitro* regeneration experiments. The zygotic embryos and pre-culture needles were then excised and used for the induction of callus.

### Induction of callus

**Figure 2:**
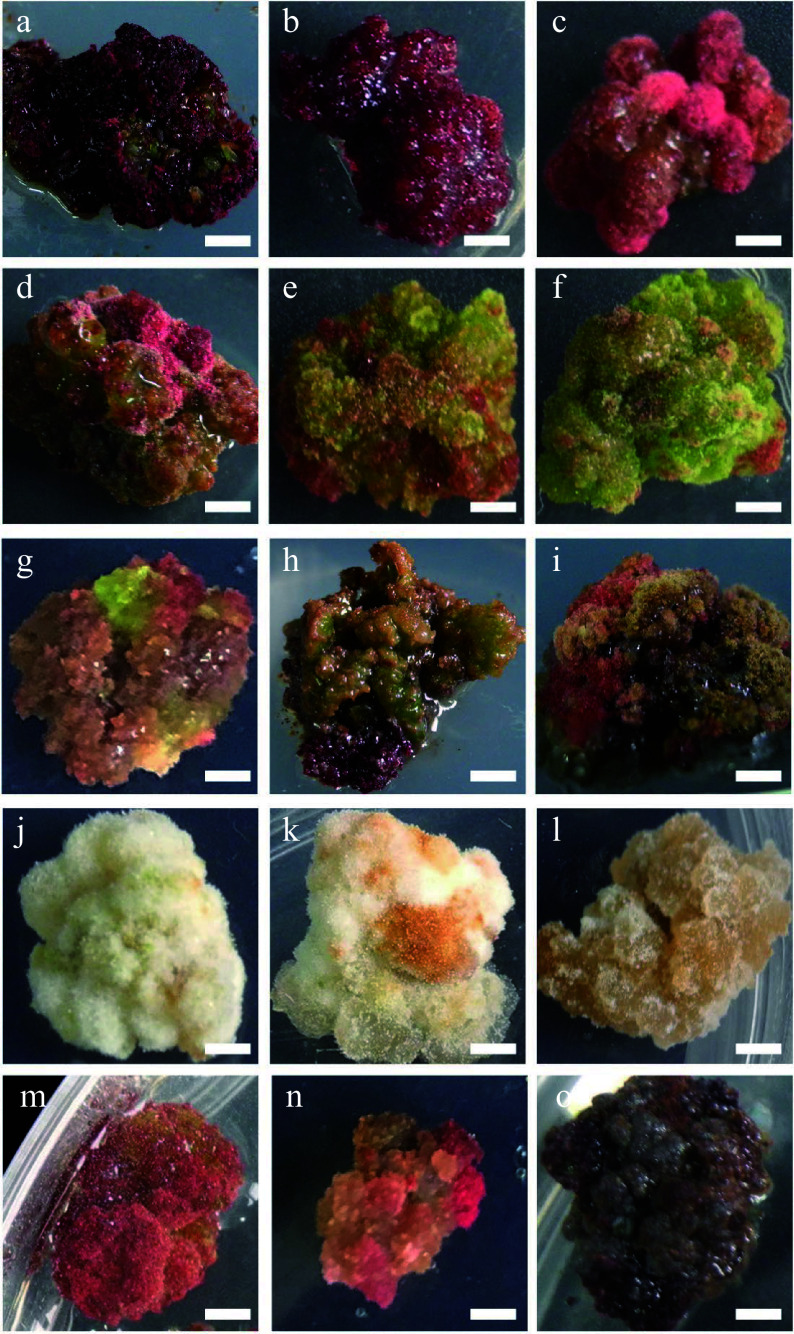
Characteristic nature of *Larix olgensis* callus from different treatments. (a) DCR + 3 mg∙L ^−1^ 6-BA + 0.3 mg∙L ^−1^ NAA (90%+). (b) DCR+2 mg∙L ^−1^ 6-BA+0.2 mg∙L ^−1^ NAA. (c) DCR + 1 mg∙L ^−1^ 6-BA + 0.1 mg∙L ^−1^ NAA. (d) DCR + 3 mg∙L ^−1^ 6-BA + 0.3 mg∙L ^−1^ NAA, a genotype different from that in (a). (e) DCR + 1 mg∙L ^−1^ 6-BA + 1 mg∙L ^−1^ NAA. (f) DCR + 0.3 mg∙L ^−1^ 6-BA + 3 mg∙L ^−1^ NAA. (g) MS + 0.5 mg∙L ^−1^ 6-BA + 0.05 mg∙L ^−1^ NAA. (h) DCR + 0.5 mg∙L ^−1^ 6-BA + 0.05 mg∙L ^−1^ NAA. (i) DCR + 0.5 mg∙L ^−1^ 6-BA + 0.05 mg∙L ^−1^ NAA transfer to DCR + 2 mg∙L ^−1^ 6-BA + 0.2 mg∙L ^−1^ NAA. (j) DCR + 0.3 mg∙L ^−1^ 6-BA + 3 mg∙L ^−1^ NAA (in dark). (k) MS+0.05 mg∙L ^−1^ 6-BA + 0.5 mg∙L ^−1^ NAA (in dark). (l) MS + 3 mg∙L ^−1^ 6-BA + 0.3 mg∙L ^−1^ NAA (in dark). (m), (n) Modified high auxin culture, the yellow callus turn red. (o) After subculture over five times, the callus turned brown. The bar in the pictures is 0.32 cm except in (n) which is 0.43 cm.

The explants (zygotic embryos and needles) were obtained in sterile environments. The endosperm was removed from the seeds and the leftover mature zygotic embryos were used directly for callus induction. The needles were collected as described above. Fifteen explants (of zygotic embryo or needles) were placed in 40 mL of callus induction medium (CIM) in a 90 mm × 20 mm crystal-grade polystyrene Petri dish (DA TANG MEDICAL INSTRUMENT) with six replicates. The zygotic embryo and needles were placed separately. The MS medium and DCR medium supplemented with cytokinins 6-BA (0.1, 0.2, 0.5, 1, 2, and 3 mg∙L ^−1^) in combination with auxin NAA at various concentrations（0.1, 0.2, 0.5, 1, 2, and 3 mg·L ^−1^） was used as the CIM. The explants in CIM were kept at 25 ± 2 °C and 70% relative humidity under white fluorescent tubes (60 μmol∙m ^−2^∙s ^−1^ light intensity) in a 16-h photoperiod system until the callus developed ( Fig. 1). The nature of the callus and the callus percentage induction were determined after 8 weeks of incubation ( Fig. 2 ).

### Regeneration of shoots

**Figure 3:**
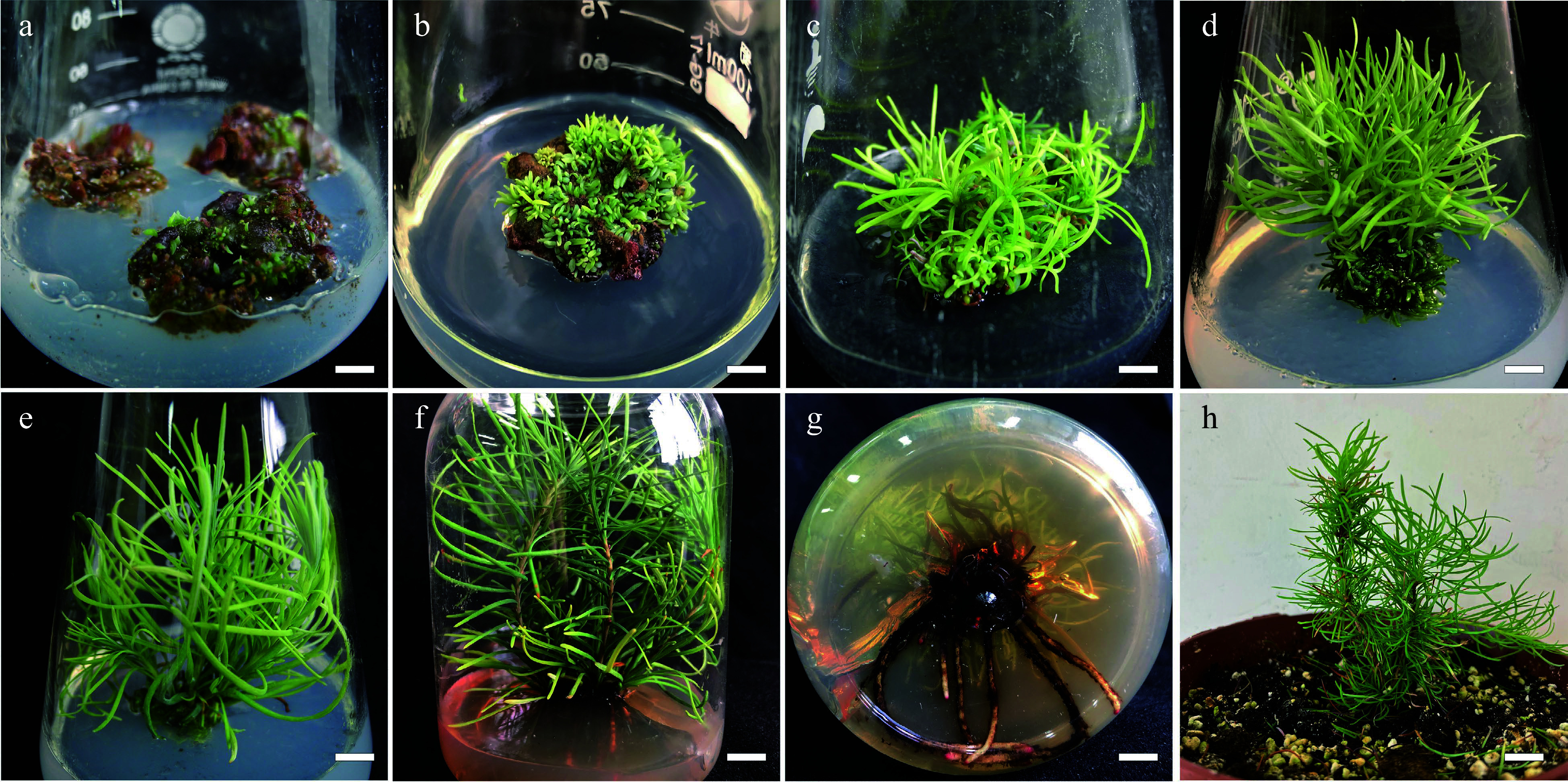
Summary of *in vitro* propagation from callus of *Larix olgensis*. (a) New shoots developed from callus placed in the DCR medium supplemented with 1.0 mg∙L ^−1^ BA and 0.1 mg∙L ^−1^ NAA, bar = 0.6 cm. (b) Shoot organogenesis occurring on callus after 8 weeks in regeneration medium, bar = 0.6 cm. (c) Developed shoots from callus, bar = 0.6 cm. (d) Adventitious shoots elongation, bar = 0.6 cm. (e) Further shoot elongation, bar = 0.68 cm. (f) Rooting of *in vitro* regenerated shoots in DCR medium supplemented with 3 mg∙L ^−1^ IBA and 100 mg∙L ^−1^ PG, bar = 0.83 cm. (g) Roots of fully developed plantlets, bar = 0.61 cm. (h) Acclimatized potted plants, bar = 2 cm.

Callus was moved to DCR medium augmented with cytokinin 6-BA (0.5, 1.0, 2.0 mg∙L ^−1^) and auxin NAA at various concentrations (0.05, 0.1, 0.2, and 0.5 mg∙L ^−1^) for shoot regeneration. Six replicates were made for each treatment, comprising 10 calli in 50 mL of the shoot regeneration medium in an Erlenmeyer flask (GG-17, 100 mL, SHUNIU). The callus cultures were kept at 25 ± 1 °C and relative humidity of 70% under white fluorescent tubes (60 μmol∙m ^−2^∙s ^−1^ light intensity) in a 16-h photoperiod system. At 2-week intervals, until shoots regenerated, the *L. olgensis* calli ( Fig. 3a) were subcultured onto fresh medium of the same composition with or without AC ( Fig. 3b). Shoots produced per callus were counted, and shoot regeneration rate was determined after 6 weeks. Regenerated *L. olgensis* shoots thereafter were moved to the medium for elongation ( Fig. 3c).

### Elongation of shoots

The regenerated *L. olgensis* shoots were cultured in the DCR medium (50 mL) supplemented with AC (0, 2 g∙L ^−1^) in addition to cytokinins 6-BA (0.05, 0.1, 0.15, 0.2, and 3 mg∙L ^−1^) and auxin NAA at various concentrations (0.005, 0.01, 0.015, 0.02 and 0.03 mg∙L ^−1^) in combination for shoot elongation in polystyrene culture vessels (ZP5-330, SHJIAFENG). Three regenerated *L. olgensis* shoots were set up in each vessel, with 20 replications for this experiment. The elongation cultures were kept at 25 ± 1 °C and relative humidity of 70% under white fluorescent tubes (60 μmol∙m ^−2^∙s ^−1^ light intensity) in a 16-h photoperiod system. At 2-week intervals, the *L. olgensis* shoots were subcultured on fresh media of the same composition ( Fig. 3d). Shoot elongation percentage (%) were counted, and elongation lengths were determined after 6 weeks. Elongated *L. olgensis* shoots thereafter were moved to the medium for rooting ( Fig. 3e).

### Rooting of regeneration shoots

The elongated *L. olgensis* shoots were cultured in DCR medium (100 mL) of various strengths (i.e. DCR, 1/2 DCR) supplemented with auxin [1-naphthaleneacetic acid (NAA) or indole-3-butyric acid (IBA)] (0.5, 1.0, 1.5, 2.0, 2.5 mg∙L ^−1^) either singly or in combination with Phloroglucinol (PG) (0, 50 , 100, 150 mg∙L ^−1^) and AC 2 g∙L ^−1^ in polystyrene culture vessels (125 mm × 110 mm). Four regenerated *L. olgensis* shoots with 20 replications for this experiment. The elongation cultures were kept at 25 ± 1 °C and relative humidity of 70% under white fluorescent tubes (60 μmol∙m ^−2^∙s ^−1^ light intensity) in a 16-h photoperiod system. Rooting rates and root numbers were determined for each treatment after culture for 10 weeks, with no subculture during rooting ( Fig. 3f, g).

### Acclimatization

After removing the medium traces from the roots of each regenerated *L. olgensis* plantlet by rinsing in running water from a tap, the plantlets were moved to a mixture of peat : organic cultivation soil : perlite (3:6:1) in 22 cm diameter plastic pots ( Fig. 3h). The plantlets were covered with transparent plastic bags ensuring adequate humidity and kept in growth chambers operating under a 16-h photoperiod (33.73 μmol∙m ^−2^∙s ^−1^ light intensity) at ~25 °C and 70% relative humidity. The polyethylene coverings were opened gradually after 3 weeks as the plantlets acclimatized. Plant survival rates were determined at 6 weeks following acclimatization.

### Determination of taxifolin in regenerated *L. olgensis* at different stages

In this study, different fresh calli and tissue of various stages were used for taxifolin content determination. Callus-1 ( Fig. 2n), callus-2 ( Fig. 2h), callus-3 ( Fig. 2a), adventitious shoots, elongation shoots, lignified elongation shoots, adventitious roots, were freeze-dried at −70 °C for 24 h. The dried tissue was ground into a powder with a mortar and sifted through 40 mesh for standby. Each sample was weighed accurately with 100 mg, and taxifolin was extracted by adding 1 mL methanol for tissue bomogenate（8,500 rpm, 4 × 15 s）and ultrasound 100 khz for 20 min. After that, the solution was centrifuged for 6 min (15,000 rpm), the supernatant was taken and diluted 10 times with methanol, and then mixed with H _2_O 1:1 for LC-MS detection.

LC-MS analysis was carried out on Waters ACQUITY I-Class (Waters Technology Shanghai, China), and Sciex Triple Quad 5500 (Sciex Shanghai, China). The injection volume of the sample was 2 µL and the column temperature was kept at 30 °C. The binary elution solvent consisted of A [0.1% Formic Acid in Methanol/Acetonitrile (1/9, v/v)] and B (0.1% Formic Acid in H _2_O): 85% : 15%, and a gradient elution procedure was used. A cosmosil column Waters HSS T3 (100 mm × 2.1 mm, 1.6 μM) was used. The flow rate was maintained at 0.5 mL·min ^−1^. The UV spectrum of taxifolin was obtained with 290 nm detection wavelength.

### Statistical analysis

All experimental data were analyzed by one-way ANOVA with Tukey's post-hoc multiple comparison tests, using SPSS (IBM SPSS Statistics 27.0). In CIM, three leaf/root segment explants with 20 replications were used. For shoot regeneration, six calli pieces were used with 10 replications, and four regenerated *L. olgensis* shoots with 20 replications were used for rooting. Means were regarded as statistically significant at *p* ≤ 0.05.

## RESULTS

### Callus induction

Among all the treatments, the highest percentage of callus induction was recorded in the explants cultivated on the DCR medium augmented with 3.0 mg∙L ^−1^ 6-BA together with 0.3 mg∙L ^−1^ NAA for both *L. olgensis* zygotic embryo (96.8%) and needle (86.7%) explants ( Table 1).

**Table 1:** Induction percentage and characteristics of callus from mature zygotic explants and needle explants of *Larix olgensis*.

Order	Basic medium	Plant growth regulators (mg∙L ^−1^)	Mature zygotic embryo explants				Needle explants		
			Callus induction rate (%)	Color	Texture		Callus induction rate (%)	Color	Texture
1	MS	6-BA 3:NAA 0.3	90.2 ± 0.11 ^a^	Rose red	Compact		43.3 ± 1.91 ^bc^	Brown	Compact
2		6-BA 2:NAA 0.2	90.1 ± 1.73 ^a^	Pink	Compact		38.9 ± 1.13 ^bcd^	Brown	Compact
3		6-BA 1:NAA 0.1	78.2 ± 4.45 ^b^	Red and white	friable		35.6 ± 2.94 ^cdc^	brown	Compact
4		6-BA 0.5:NAA0.05	52.1 ± 1.41 ^cd^	Red and white	Friable		27.8 ± 1.11 ^fg^	Brown	Friable
5		6-BA 1: NAA 1	51.6 ± 1.86 ^cd^	Cream	Compact		43.3 ± 0 ^bc^	Cream	Friable
6		6-BA 0.05:NAA 0.5	32.2 ± 1.68 ^f^	Cream	Friable		38.9 ± 111 ^bcd^	Yellow and green	Friable
7		6-BA 0.1:NAA 1	43.4 ± 3.41 ^de^	Yellow and green	Friable		27.8 ± 1.11 ^fg^	Yellow and green	Friable
8		6-BA 0.3:NAA 3	44.5 ± 3.26 ^de^	Yellow and green	Friable		13.3 ± 1.93 ^h^	Yellow and green	Compact
9	DCR	6-BA 3:NAA 0.3	96.8 ± 1.86 ^a^	Rose red	Compact		86.7 ± 1.93 ^a^	Rose red	Compact
10		6-BA 2:NAA 0.2	92.2 ± 0.97 ^a^	Rose red	Compact		46.7 ± 1.93 ^b^	Green	Friable
11		6-BA 1:NAA 0.1	76.1 ± 1.94 ^b^	Pink	Friable		22.2 ± 1.11 ^gh^	Yellow and green	Friable
12		6-BA 0.5:NAA0.05	49.0 ± 5.35 ^cd^	Pink	Friable		14.55 ± 2.22 ^h^	Dark brown	Friable
13		6-BA 1: NAA 1	51.2 ± 2.46 ^cd^	Red and white	Compact		32.2 ± 1.11 ^efg^	Green	Compact
14		6-BA 0.05:NAA 0.5	36.6 ± 3.51 ^ef^	Red and white	Friable		34.5 ± 7.78 ^cde^	Cream	Friable
15		6-BA 0.1:NAA 1	50.1 ± 2.50 ^cd^	Red and white	Compact		32.2 ± 8.89 ^efg^	Yellow and green	Compact
16		6-BA 0.3:NAA 3	55.7 ± 4.93 ^c^	Cream	Compact		23.3 ± 0 ^gh^	Yellow and green	Compact
Means ( ± standard error) within a column followed by the same superscript letter are not significantly different using Tukey’s multiple comparison test and *p* ≤ 0.05.									

Although in the about-mention medium both the zygotic embryo explants and needle explants could achieve the highest percentage of callus induction rate, the two type of explants responded significantly differently in other treatments. For zygotic embryo explants, there are no significant differences (in the callus induction rate) from the highest percentage callus in MS media containing 3.0 mg∙L ^−1^ 6-BA and 0.3 mg∙L ^−1^ NAA (90.2%), 2.0 mg∙L ^−1^ BA and 0.2 mg∙L ^−1^ NAA (90.1%), and DCR media augmenting with 3.0 mg∙L ^−1^ 6-BA and 0.3 mg∙L ^−1^ (96.8%), 2.0 mg∙L ^−1^ BA and 0.2 mg∙L ^−1^ NAA (90.1%) among others ( Fig. 1e− h, Table 1).

The highest percentage of callus induction from the needle explants was 86.7% in the DCR medium augmented with 3 mg∙L ^−1^ 6-BA and 0.3 mg∙L ^−1^ NAA, which was significantly higher ( *p* ≤ 0.05) than that in the other treatments ( Fig. 1a− d, Table 1). The lowest percentage of induced callus from *L. olgensis* zygotic embryo explants was 32.3% in the MS medium augmented with 0.05 mg∙L ^−1^ 6-BA together with 0.5 mg∙L ^−1^ NAA, and that from needle explants were in the DCR medium augmented with 0.3 mg∙L ^−1^ 6-BA together with 0.3 mg∙L ^−1^ NAA (13.3%).

The effect of plant growth regulators (PGRs) combination was tested. The ratio of auxin and cytokinin of 1/10 showed a better callus induction response than that of 1/1 and 10/1 ( Table 1). Once the ratio was determined, it was found that the callus induction rate was increased along with the promotion in the concentration of the PGRs combination. Meanwhile, the suitable basic mediums of callus induction from mature zygotic embryos were MS and DCR, while needle explants preferred DCR basic medium.

The calli produced from both zygotic embryos and needle explants had different textures and colors. These colors were pink ( Fig. 2a− d), green ( Fig. 2e, f), cream ( Fig. 2g − i), or dark brown ( Fig. 2o), etc ( Fig. 2g− i, m, n), and their textures were either compact or friable depending on the medium composition and explant type ( Table 1, Fig. 2). Furthermore, the compact rose red callus is the best for shoot regeneration ( Fig. 2a, b).

### Shoot regeneration

For the zygotic explants, we found that the MS medium augmented with 1.0 mg∙L ^−1^ 6-BA and 0.1 mg∙L ^−1^ NAA had the highest shoot regeneration rate (83.3 ± 1.93% and 528 ± 11.5 number of shoots per callus), followed by the medium supplemented with 1.0 mg/L 6-BA in addition to 0.2 mg∙L ^−1^ NAA (76.7 ± 1.93%) shoot regeneration rate with the highest shoot number per callus (636 ± 21.7) ( Table 2, Fig. 3a).

**Table 2:** Percentage shoot regeneration from calli of *Larix olgensis*.

				Mature zygotic embryo explants			Needle explants	
	Plant growth regulators (mg∙L ^−1^)			Percentage shoot regeneration (%)	Average number of adventitious shoots		Percentage shoot regeneration (%)	Average number of adventitious shoots
	6-BA	NAA						
1	0.5	0.05		41.1 ± 2.94 ^fg^	304 ± 7.5 ^d^		32.22 ± 1.11 ^de^	120 ± 6.1 ^h^
2	0.5	0.1		42.2 ± 1.11 ^fg^	315 ± 4.5 ^d^		32.22 ± 2.22 ^de^	111 ± 3.8 ^h^
3	0.5	0.2		32.2 ± 1.11 ^h^	150 ± 14.8 ^f^		28.89 ± 1.11 ^e^	66 ± 2.3 ^i^
4	0.5	0.5		40.0 ± 1.93 ^g^	211 ± 5.5 ^e^		34.44 ± 1.11 ^d^	51 ± 1.8 ^g^
5	1	0.05		57.8 ± 1.11 ^d^	426 ± 13.9 ^c^		68.89 ± 1.11 ^a^	161 ± 6.2 ^f^
6	1	0.1		83.3 ± 1.93 ^a^	528 ± 11.5 ^b^		73.33 ± 1.93 ^a^	307 ± 1.8 ^b^
7	1	0.2		76.7 ± 1.93 ^b^	636 ± 21.7 ^a^		72.22 ± 1.11 ^a^	323 ± 3.8 ^a^
8	1	0.5		65.6 ± 2.22 ^c^	460 ± 22.8 ^c^		73.33 ± 1.93 ^a^	221 ± 6.4 ^c^
9	2	0.05		53.3 ± 3.85 ^de^	322 ± 11.7 ^d^		50.00 ± 1.93 ^c^	176 ± 5.5 ^e^
10	2	0.1		47.8 ± 2.94 ^ef^	237 ± 11.5 ^d^		51.11 ± 1.11 ^c^	141 ± 7.0 ^g^
11	2	0.2		46.7 ± 1.93 ^efg^	423 ± 23.5 ^c^		57.78 ± 2.22 ^b^	203 ± 3.9 ^d^
12	2	0.5		47.8 ± 1.11 ^ef^	200 ± 6.1 ^e^		33.33 ± 1.93 ^de^	57 ± 1.2 ^ig^
Means (± standard error) within a column followed by the same superscript letter are not significantly different using Tukey’s multiple comparison test and *p* ≤ 0.05.								

Meanwhile, for the needle explants, the highest percentage of regeneration (73.3 ± 1.93%) and the number of shoots per callus (307 ± 1.8) were recorded in the explants cultivated on the DCR medium augmented with 1.0 mg∙L ^−1^ 6-BA, 0.1 mg∙L ^−1^ NAA, and 0.1 mg∙L ^−1^ TDZ, followed by the results in the medium supplemented with 1.0 mg∙L ^−1^ 6-BA in addition to 0.2 mg∙L ^−1^ NAA (with shoot regeneration percentage and the number of shoots per callus, 72.22 ± 1.11% and 323 ± 3.8, respectively) ( Table 2 *,* Fig. 3b). The highest percentage of regeneration (73.3 ± 1.93%) was also recorded in the medium supplemented with 1.0 mg∙L ^−1^ 6-BA in addition to 0.5 mg∙L ^−1^ NAA, but the number of shoots per callus (221 ± 6.4) was significantly lower than that in medium 6.

Both shoot regeneration percentage and shoot number per callus were generally higher in media supplemented with cytokinin (BA) in combination with auxin (NAA), which ratio ranges from 10/1 to 5/1. In addition, medium 2-6 and medium 2-7, 2-1, 2-2, and 2-11 also showed relatively high induction rates. Although the differences in shoot regeneration percentage were not statistically significant, medium supplemented with 6-BA in combination with higher NAA were generally associated with a low number of shoots induced from each callus on average ( Table 2 *,* Fig. 3). If the ratio of 6-BA to NAA is fixed, with the increase of the PGRs concentration, the shoot regeneration percentage and the number of shoots per callus showed an upward trend initially and then declined.

Due to the limited callus size and the number of the subculture of needles, the number of shoots per callus induced from needles was lower than that from zygotic embryos, but there is no significant difference in the shoot regeneration percentage between zygotic embryos and needles.

### Shoot elongation

The zygotic embryo explants and the needle explants were inoculated in the same shoot regeneration culture medium for 4 weeks before subculture to the elongation treatment. The DCR medium augmented with 0.5 mg∙L ^−1^ BA, 0.05 mg∙L ^−1^ NAA, and 2 g∙L ^−1^ AC achieved the highest elongation percentage of shoots (75.6 ± 2.94%) and the longest average shoot length (3.5 ± 0.11 cm). The percentage shoot elongation and average shoot length significantly differed ( *p* < 0.05) from that in DCR without any AC (control) ( Table 3, Fig. 3c− e). In the same medium without AC, the percentage of shoot elongation and average shoot length were 65.2 ± 1.11% and 1.6 ± 0.11 cm, respectively. Compared medium 3-2 (65.6 ± 1.11%, 1.6 ± 0.06 cm) to medium 3-3 (75.6 ± 2.92%, 3.5 ± 0.11 cm), medium 3-4 (46.7 ± 1.93%, 1.3 ± 0.03 cm) to medium 3-5 (61.1 ± 1.11%, 2.8 ± 0.12 cm), medium 3-6 (21.1 ± 1.11%, 1.4 ± 0.01 cm) to medium 3-7 (35.6 ± 2.22%, 2.2 ± 0.1 cm), it was clear that AC significantly increased the percentage of shoots elongation and average shoot length.

**Table 3:** Effect of different concentrations of 6-BA and NAA on adventitious bud elongation of *Larix olgensis*.

	Plant growth regulators and AC (mg∙L ^−1^)	Adventitious shoot elongation percentage (%)	Average shoot length (cm)
1	6-BA 1:NAA 0.1	21.1 ± 2.22 ^e^	0.9 ± 0.03 ^e^
2	6-BA 0.5:NAA 0.05	65.6 ± 1.11 ^b^	1.6 ± 0.06 ^d^
3	6-BA 0.5:NAA 0.05:AC 2000	75.6 ± 2.94 ^a^	3.5 ± 0.11 ^a^
4	6-BA 0.3:NAA 0.03	46.7 ± 1.93 ^c^	1.3 ± 0.03 ^d^
5	6-BA 0.3:NAA 0.03:AC 2000	61.1 ± 1.11 ^b^	2.8 ± 0.12 ^b^
6	6-BA 0.1:NAA 0.01	21.1 ± 1.11 ^e^	1.4 ± 0.01 ^d^
7	6-BA 0.1:NAA 0.01:AC 2000	35.6 ± 2.22 ^d^	2.2 ± 0.10 ^c^
Means (± standard error) within a column followed by the same superscript letter are not significantly different using Tukey’s multiple comparison test and *p* ≤ 0.05.			

The elongation culture of *L. olgensis* was DCR medium supplemented with a certain ratio but different concentrations of BA and NAA. According to Table 3, a comparison of the elongation rate and average shoot length among medium 3-1 (21.2 ± 2.22%, 0.9 ± 0.03 cm), medium 3-2 (65.6 ± 1.11%, 1.6 ± 0.06 cm), medium 3-4 (46.7 ± 1.93%, 1.3 ± 0.03 cm), which depicted lower concentrations of the PGRs promoted the shoot elongation, but when it reduced to a certain level, poor elongation also resulted.

### Rooting

In our study, the first regeneration plantlet with roots (1−2 mm) was seen on the 38 ^th^ day in DCR medium supplemented with 3 m∙L ^−1^ IBA and 100 mg∙L ^−1^ PG, and the highest adventitious root induction rate was 62.2 ± 5.88% ( Table 4, Fig. 3f, g). Even if the concentration of auxin is continuously increased, the single application of auxin has little effect on rooting. The rooting rates of DCR medium supplemented with 3 mg∙L ^−1^ of IBA or NAA were 13.3%, and 8.9 ± 2.22%, respectively. But in 1/2 DCR medium with the same PGRs were 11.11 ± 2.22%, and 15.56 ± 2.22%, respectively. Rooting in these mediums took at least 60 d.

**Table 4:** Rooting of regenerated shoots in DCR media supplemented with auxin, AC and PG strength.

Basic medium	Exogenous additives (mg∙L ^−1^)	Adventitious root induction percentage (%)	Rooting start time (d)
DCR	NAA 3	8.9 ± 2.22 ^e^	70
	IBA 2:NAA 2	15.6 ± 2.22 ^de^	64
	IBA 3:AC 2000	46.7 ± 3.85 ^b^	52
	IBA 3	13.3 ± 0.00 ^e^	63
	IBA 3:PG 50	33.3 ± 3.85 ^c^	45
	IBA 3:PG 100	62.2 ± 5.88 ^a^	38
	IBA 3:PG 150	57.8 ± 4.44 ^ab^	36
	IBA 3:PG 100:AC 2000	53.3 ± 3.85 ^b^	32
1/2DCR	NAA 3	15.56 ± 2.22 ^de^	70
	IBA 2:NAA 2	22.22 ± 2.22 ^d^	63
	IBA 3:AC 2000	35.56 ± 2.22 ^c^	60
	IBA 3	11.11 ± 2.22 ^e^	60
	IBA 3:PG 50	31.11 ± 4.44 ^c^	50
	IBA 3:PG 100	48.89 ± 2.22 ^b^	41
	IBA 3:PG 150	46.67 ± 0 ^b^	40
	IBA 3:PG 100:AC 2000	37.78 ± 2.22 ^c^	36
Means (± standard error) within a column followed by the same superscript letter are not significantly different using Tukey’s multiple comparison test and *p* ≤ 0.05.			

The addition of AC and PG promoted root formation to a large extent and significantly increased the rooting percentage. In the medium supplemented with PG, once the root primordium is produced, the adventitious roots were produced along the main stem, and the roots were quickly formed to produce a strong root system ( Fig. 3g). The concentration of PG significantly affected the rooting rate in both 1/2 DCR and DCR medium. Specifically, among the three concentrations of PG tested, 50 mg∙L ^−1^, 100 mg∙L ^−1^, and 150 mg∙L ^−1^, the 100mg∙L ^−1^ PG had a better effect on rooting (Supplemental Fig. 1a−d). The root system from the medium supplemented with AC was slender, and lateral roots were produced in prolonged culture (Supplemental Fig. 1e, f).

### Acclimatization

After 6 weeks of acclimatization, the *in vitro* regenerated *L. olgensis* plantlets showed a high survival rate of 90%. The acclimatized *L. olgensis* plantlets grew well and displayed normal growth characteristics and morphology typical of the plant species ( Fig. 3h).

### Determination of taxifolin content in regenerated *L. olgensis* tissues at different stages

To determine the taxifolin content in different tissues and stages of *L. olgensis* plants, callus of different stages (callus-1, callus-2, callus-3), adventitious shoots, elongation shoots, lignified elongation shoots, adventitious roots, were selected and analyzed by HPLC (Supplemental Fig. 2). The result indicated that the taxifolin content in different tissues and different stages of the regenerated *L. olgensis* plants varied significantly. The content of the taxifolin in callus-1 was 1.99 µg∙g ^−1^, callus-2 was 3.9 µg∙g ^−1^, callus-3 was 5.26 µg∙g ^−1^, and in adventitious shoots, the content of the taxifolin was 4.8µg/g, while in adventitious roots was 2.86 µg∙g ^−1^. The accumulation of the taxifolin in elongation shoots and lignified elongation shoots was 28.6 µg∙g ^−1^ and 53 µg∙g ^−1^ respectively, much higher than that in other tissues.

Meanwhile, the results showed that calli in different colors and textures might affect the accumulation of taxifolin. For example, the rose-red calli accumulated more taxifolin than the calli of two other colors. The result also illustrated that the development of vascular tissue was beneficial to the accumulation of taxifolin since in the elongation shoots, the content of taxifolin was much higher. Compared with the un-lignified elongation shoots, the content of taxifolin in the lignified shoots nearly doubled. Therefore, *in vitro* regeneration is an efficient and quick method to produce secondary metabolites.

## DISCUSSION

Due to the current large demand for larch timber, the supply of seedlings and the establishment of plantations have become very urgent. *In vitro* regeneration technology provides an efficient way for large numbers of seedling production in a short time and has been widely used in large-scale propagation of high-quality seedlings, genetic transformation, and gene editing. The *L. olgensis* regeneration system is set up in this study, the process is stable and reliable, and not easily affected by external conditions, and therefore, is highly controllable and annual production can be assured.

The research on the *in vitro* regeneration system of larch mainly contains somatic embryogenesis and organogenesis. Current research on *in vitro* regeneration of larch mainly focuses on inducing embryogenic callus that leads to somatic embryogenesis. The induced somatic embryos have characteristics similar to mature zygotic embryos and can directly generate stems and roots through suitable culture. However, some research showed that the germination rate of the induced somatic embryos was uncontrollable ^[ 6] ^, and the malformed embryos accounted for a large proportion. Some believe that somatic embryos are more suitable for cryopreservation and production of artificial seeds ^[ 23] ^, but the subsequent growing time is the same or even longer than that of larch seedlings from natural seeds. In this study, mature embryos are regenerated via the callus, and under suitable culture conditions, more calli can be subcultured, and more adventitious shoots can subsequently be differentiated. Since a large amount of biomass can be produced under certain culture conditions, the growth rate and development direction can be adjusted by using different culture conditions. Tissue products in various culture stages can also be used for active ingredient extraction. The *in vitro* regeneration system of larch was optimized in the following aspects.

Different species respond to *in vitro* regeneration quite differently, which might be the reflection of differences in nutrient absorption. It is crucial for species to confirm a rationally basic medium. Classical MS medium with a high nutrient concentration of inorganic salts is favored in plant tissue culture ^[ 24] ^, especially in the cultivation of herbaceous plants, such as cornflower ^[ 25]^ ( *Gerbera jamesonii*), lily ( *Lilium orientalis*) ^[ 26] ^, andrographis ( *Andrographis alata*) ^[ 27] ^, chandelier flowers ( *Ceropegia mohanramii*) ^[ 28]^ *,* etc. Generally, researchers believe that at present, the regeneration of woody plants is more difficult than that of herbaceous plants ^[ 29] ^. It is shown that the absorption capacity of the basic medium is different for different life-form plants, and sometimes the MS medium does not yield good results in some woody plants. For example, for the conifer juniper ^[ 30]^ ( *Juniperus* L.), researchers gradually replaced MS with WPM medium during the culture. The researchers used a modified MS medium with half-strength salt and reduced the concentration of KNO _3_ in the medium at a later stage for a good culture effect. The study by Samiei et al. ^[ 31]^ suggested that Van der Salm (VS) modified by MS medium with reduced inorganic salts has a better effect than MS in culturing *Rosa canina*. In the cultivation of *Fagaceae* chestnut ( *Castanea sativa* × *Castanea mollissima*) ^[ 32] ^, researchers used MS medium with reduced salt concentration, combined with WPM, to obtain stable chestnut regeneration seedlings. The macroelement salt ion molar concentration of MS is about three times that of WPM and nine times that of DCR medium. Researchers who used MS medium to cultivate the plantlets have so far not achieved efficient results in larch. In our research, the lower inorganic salt ion concentration DCR basic medium is suitable for *L. olgensis* subsequent development. The results are consistent with the above mentioned reports ( Table 1, Fig. 1).

Many studies have shown that the use of single plant growth regulator has a limited effect on callus tissue during larch regeneration. In this study, we investigated the effects of auxin and cytokinin in different ratios and the strength of the combined concentration on various processes in the regeneration of mature zygotic embryos and needles. The dominant plant growth regulator and the ratios of different PGRs both played crucial roles in callus induction. The combination and concentration of hormones for *in vitro* regeneration of mature zygotic embryos and needles were determined. In the *in vitro* regeneration of plants, the combined use of cytokinins and auxins can influence the growth direction of the materials. The combination of cytokinin 6-BA and auxin NAA in different concentration groups were used to study the concentration ratio and intensity of growth regulators required in each stage of larch development. It was found that in the stage of callus induction of mature zygotic embryos and needles, larch needs a higher concentration of cytokinin, thus forming a large number of calli. When the 6-BA/NAA ratio is 10, it is beneficial to promote callus induction. At this ratio, increasing the concentration to three times (taking 6-BA as 1 mg∙L ^−1^ as an example) can accelerate the formation of calli. However, under the condition of high auxin, callus quality is poor and consequently difficult to differentiate ( Table 1, Fig. 2l, o). A shorter subculture cycle may be beneficial for callus induction under high auxin culture conditions.

Our study suggests that the adventitious shoots subsequently differentiate from callus and should require a lower concentration of cytokinin in larch ( Fig. 3). The method was considered to be effective to obtain regenerated plantlets. In addition, adding a certain amount of activated carbon is conducive to the elongation of larch ( Table 3). In contrast, in the regeneration of Ash ( *Fraxinus mandshurica*) in 2020 ^[ 33] ^, using long-term and high concentration phytohormone cultivation, the number of adventitious bud differentiation is extremely low, and complete plants cannot be obtained. The results of elm trees ( *Ulmus glabra* and *Ulmus laevis*) *in vitro* regeneration also showed ^[ 34]^ that 0.5 mg∙L ^−1^ 6-BA was appropriate for plant regeneration and stem differentiation. In some ranges, both the broad-leaved tree and conifer maybe have similar responses to adventitious shoots differentiation.

Among all the tissues differentiated, the content was high in the lignified elongation shoot and the green elongation shoot. Moreover, the states of the tissues influenced the content of taxifolin. For example, the rose-red calli accumulated more taxifolin than the calli of the two other colors. It can be referred that the development of vascular tissue was beneficial to the accumulation of taxifolin since in the lignified shoots, the content of taxifolin was much higher (Supplemental Fig. S2). The result is consistent with natural larch, which also proves the potential of active ingredient production with artificial regulation ^[ 35] ^. Overall, it is obvious that *in vitro* regeneration is an efficient and quick method to produce secondary metabolites.

The *in vitro* rooting of larch is very difficult. In this study, we established a rooting system for larch. Previously, there is no effective rooting method for larch *in vitro* regeneration, or the rooting process is complicated ^[ 36] ^. Induction of adventitious roots is the most difficult step in the *in vitro* regeneration of larch, with unstable rooting and a low induction rate of adventitious roots. In the process of adventitious shoot rooting, single application of auxin was not good ( Table 4), increasing the concentration of auxin to 3 mg∙L ^−1^, or two auxins NAA and IBA in combination, the rooting effect did still not work well. However, the addition of exogenous substances such as PG and AC in combination with auxin has a good effect on rooting. In this study, the combination of 100 mg∙L ^−1^ PG and IBA obtained a good rooting effect ( Fig. 3, Supplemental Fig. S1), indicating that PG is a good exogenous additive for inducing rooting.

Above all, an efficient and complete organogenesis regeneration system was established for the first time, which would greatly benefit larch plantation. This protocol can be used for large-scale propagation of high-quality seedlings, genetic transformation, gene editing, and *in vitro* production of raw materials in various industries. Furthermore, it is a reliable reference for *in vitro* regeneration in recalcitrant species.

## CONCLUSIONS

In this study, we established an efficient and complete regeneration system for larch organogenesis regeneration for the first time, especially from the needle explants. Effects of combination of auxin and cytokinin in different ratios and different intensities on regeneration were investigated. Furthermore, we firstly reported the taxifolin accumulation and content in the different larch tissues. To the best of our knowledge, this is the first study to develop an efficient indirect regeneration protocol for *L.* *olgensis*, which can be used for large-scale breeding of high-quality seedlings, genetic transformation, and gene editing and offers a basis for the production of raw materials in various industries. It is also a reliable reference for *in vitro* regeneration in recalcitrant species.

## SUPPLEMENTARY DATA

Supplementary data to this article can be found online.

## Supplementary Information

accompanies this paper at ( https://www.maxapress.com/article/doi/10.48130/FR-2023-0004)
