# Supplementary figures and images for: Efficient organogenesis and taxifolin production system from mature zygotic embryos and needles in larch
Source: For Res (Fayettev). 2023 Feb 23;3:4. doi: 10.48130/FR-2023-0004 (PMC11524243; doi:10.48130/FR-2023-0004)

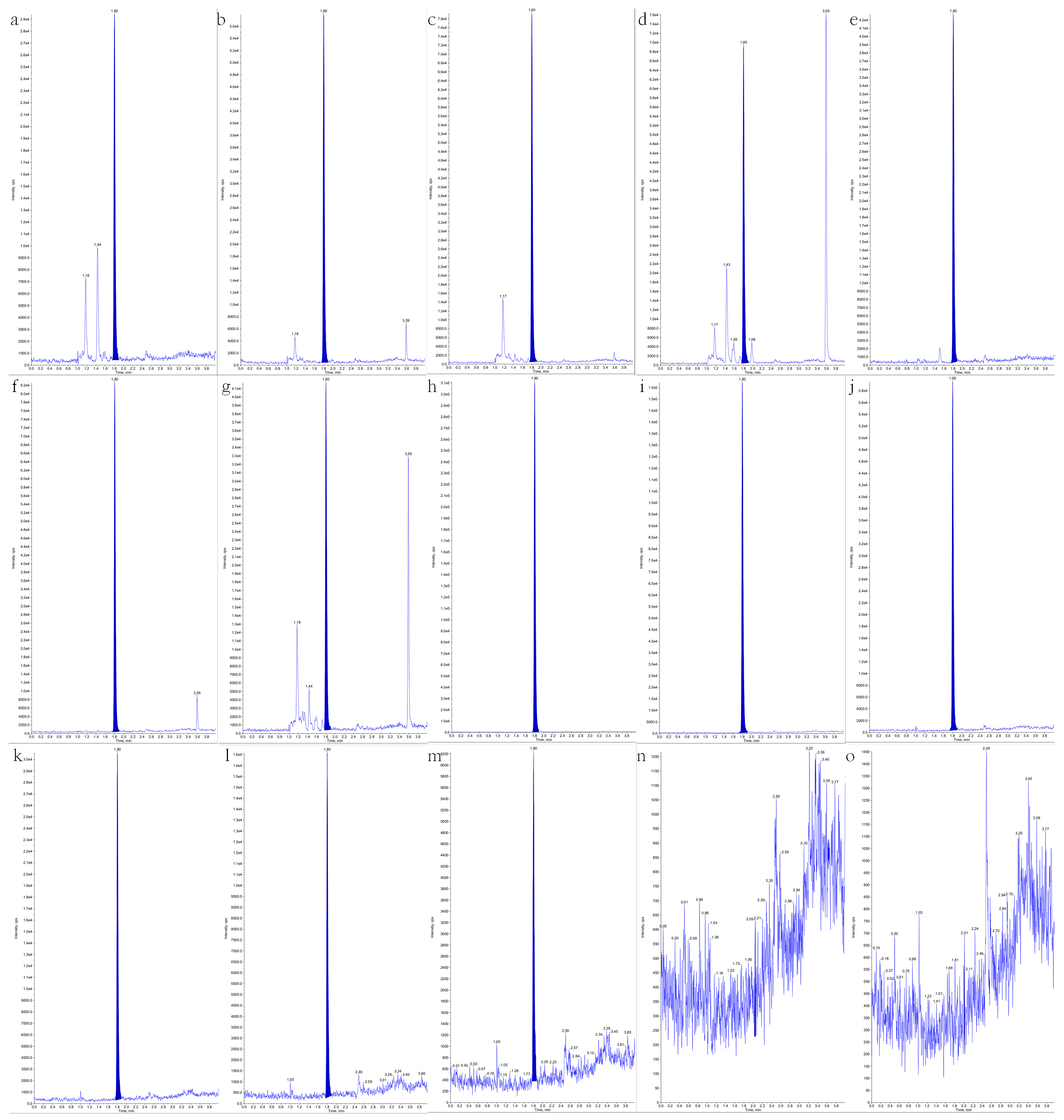

Supplement: Supplementary file 1 — Supplementary data to this article can be found online. [file FR-2023-0004-S1.zip › 10.48130_FR-2023-0004-Suppl-FigureS2.jpg]

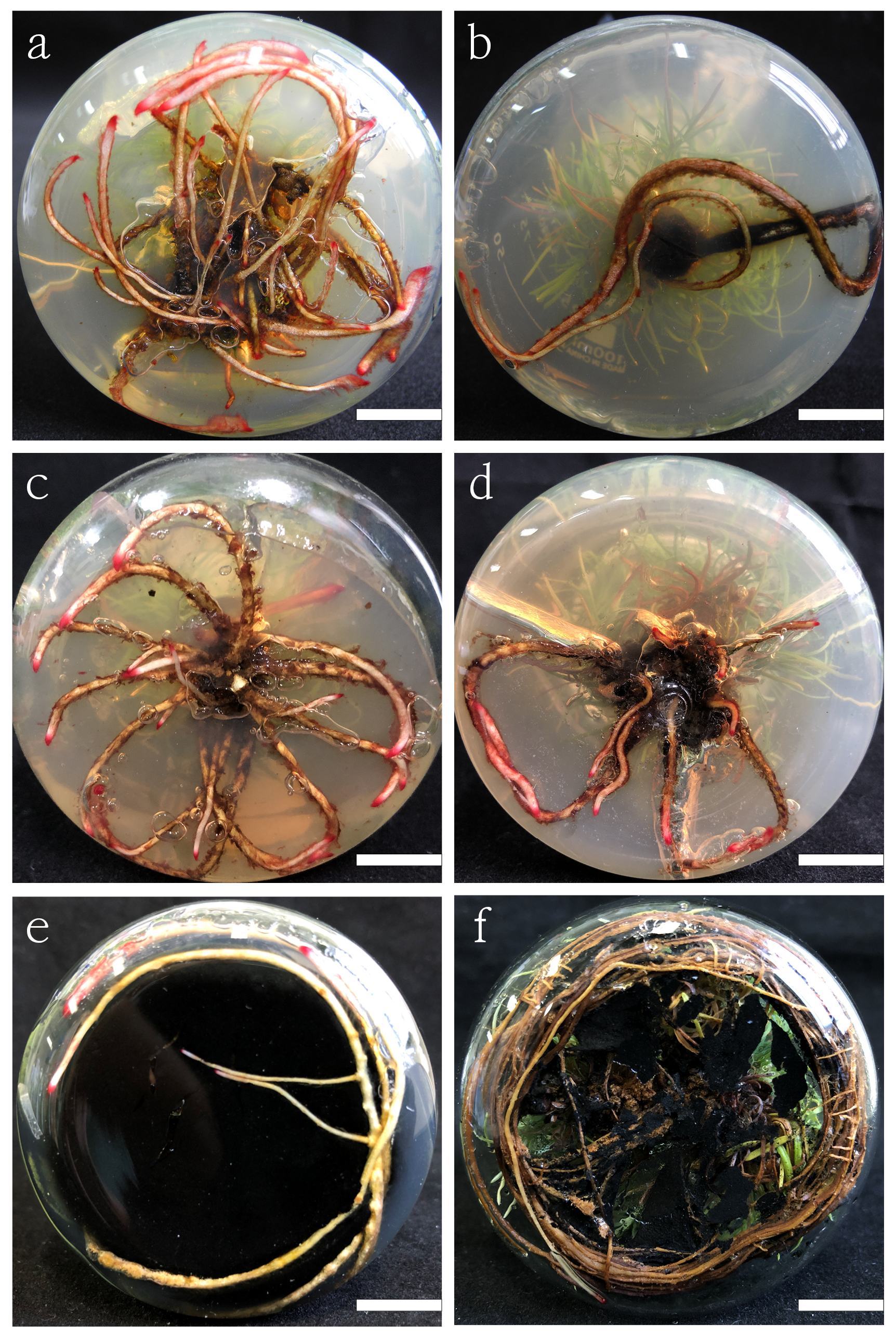

Supplement: Supplementary file 1 — Supplementary data to this article can be found online. [file FR-2023-0004-S1.zip › 10.48130_FR-2023-0004-Suppl-FigureS1.jpg]
